# Using Artificial Intelligence to Quantify Sexual Dimorphism in Aesthetic Faces: Analysis of 100 Facial Points in 42 Caucasian Celebrities

**DOI:** 10.1093/asjof/ojad046

**Published:** 2023-06-05

**Authors:** Alice S Liu, Cristina A Salinas, Basel A Sharaf

## Abstract

**Background:**

Sexual dimorphism has been studied in the faces of average populations and worldwide celebrities; however, a focused analysis of attractive Caucasian faces has not been conducted.

**Objective:**

The study harnesses the power of artificial intelligence (AI) to efficiently analyze these facial patterns in attractive Caucasian male and female celebrities.

**Methods:**

Twenty-one male and 21 female Caucasian celebrities were selected based on popular editorial rankings, modeling agencies, and casting directors from 2017 to 2022. Frontal photographs of celebrities aged 23 to 42 without facial animation were selected. One hundred facial landmarks were identified using semi-automatic image analysis software consisting of modified Apple Vision (Cupertino, CA) machine-learning algorithms with additional custom landmarks. Measurements were converted to absolute distances by fixing subjects’ white-to-white corneal diameters to the validated average in Caucasians.

**Results:**

Attractive females had significantly greater upper and middle facial proportions, more uniformly divided facial thirds, and greater canthal tilt compared with males. Attractive males had significantly greater facial height, bizygomatic and bigonial widths, medial and total brow lengths, and alar width than females. The golden ratio (1.618) was observed in the ratio of facial height to bigonial width in females (1.613), and attractive males closely approximated that ratio (1.566). There were no significant differences in interpupillary distances, eyebrow angles, or horizontal palpebral fissure lengths. No faces in either sex exhibited scleral show.

**Conclusions:**

The study is the first to utilize AI in quantifying key sexual dimorphisms among Caucasian celebrity faces. Identifying these contemporary patterns may provide valuable considerations in planning facial aesthetic and gender affirmation surgery.

**Level of Evidence: 3:**

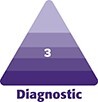

Facial aesthetics plays an important role in a variety of social outcomes ranging from social interactions, mating preferences to job hiring decisions. Magazines, social media, and television screens are filled with attractive faces, and celebrities are often the ones who receive the most public attention on these platforms and thus tend to influence beauty norms.^1^ However, the standards of an aesthetically attractive face are different for males and females, as sexual dimorphism (ie, masculinity or femininity) influences perception of facial beauty.^2^ Quantitative research has found sexually dimorphic feature differences in faces of the average populations as well as worldwide celebrities.^2-5^ However, given the variations in facial features among different ethnic groups,^6,7^ more focused analyses of attractive faces within groups are necessary. Given the influence of celebrities on society's beauty ideals and trends, identifying patterns in male and female celebrity facial features within an ethnic group may provide valuable insight in planning aesthetic facial rejuvenation as well as facial gender affirmation surgeries (FGASs).

Traditionally, obtaining facial measurements involves the use of devices such as calipers by trained personnel or manually measuring landmarks on images containing reference scale rulers. However, this is time-intensive and logistically challenging, requiring subjects to be physically in the presence of trained personnel or requiring laborious manual measurements. To improve this, we have developed a method that employs artificial intelligence (AI) found in readily available facial recognition software to assess facial landmark measurements on frontal photographs. The utility of AI in facial landmark detection lies in its speed of analysis, continual algorithmic improvements over time, and two-dimensional (2D) facial recognition has reported very high rates of accuracy.^8^ This allows us to use frontal facial photographs on subjects whom we may not be able to physically measure, such as celebrities, to perform detailed facial analysis. Studies have already shown the utility of commercially available AI facial recognition software in medical and surgical applications, such as quantifying facial paralysis and quantifying aesthetic surgical outcomes preoperative and postoperative facial emotions.^9,10^ Our study applies similar facial recognition technology; however, we have expanded and customized our technology to analyze sets of soft-tissue landmarks on frontal photographs, from which we are able to extract a myriad of observations such as landmark distances, angles, and ratios. Furthermore, our method does not require uploading subject photographs to online servers, thereby allowing potential expansion to patients in the future under Health Insurance Portability and Accountability Act compliance. To demonstrate the applications of this method, we have conducted an analysis of sexual dimorphism in Caucasian celebrities. To our knowledge, this is the first study that incorporates AI in studying facial anthropometric measurements and ratios in attractive celebrity male and female faces.

## METHODS

Twenty-one female and 21 male celebrities were selected from People magazine's “A Look Back at the Covers—Most Beautiful People in the World” issue (2022), “All the Sexiest Men Alive Covers” 2022 issue, and “The World's Highest Paid Models” 2017 issue. These lists were compiled using submissions from readers, fellow staffers and casting directors, and modeling agencies. Since facial features can differ between ages and ethnic groups, only Caucasian celebrities aged 23 to 42 were included in this study. The sample size calculation for this study was based on a validated *t*-test for differences in average bony mandibular width between males (9.35 ± 0.57) and females (8.70 ± 0.56).^3^ A study with a power of 95% would require a total sample of 18 subjects. Our study included 21 males and 21 females.

To mitigate any discrepancies in photographic standards or potential digital manipulation, all photographs included in the analysis were reviewed by 3 independent graders, including the senior author (B.A.S.). We utilized photograph sources, such as Getty Images (Seattle, WA), Shutterstock (New York, NY), and Alamy (Oxfordshire, UK) to select many of the photographs. Most photographs utilized in this study were from formal entertainment events and taken by professional photographers present at the events, as stated in the photograph credits. Images of these celebrities were used if they met the following inclusion criteria: full-face, front-view photograph, no significant cosmetic procedure, visible hairline and facial contour, and minimal facial animation.

**Figure 1.**
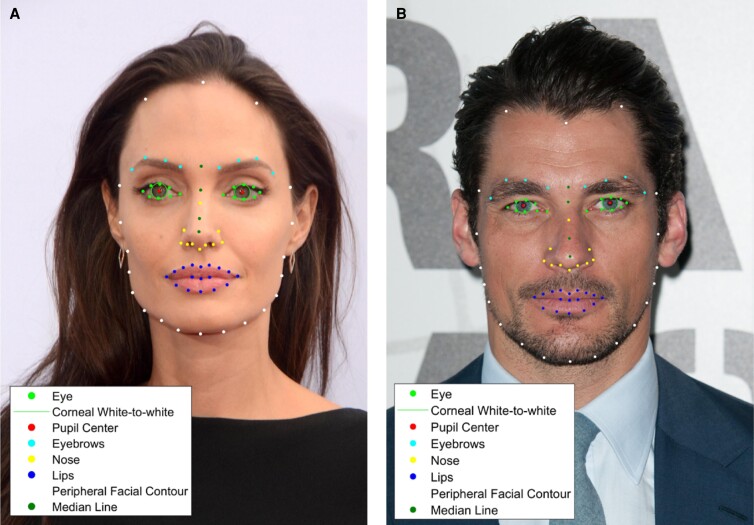
Sample subset of facial landmarks for (A) Angelina Jolie and (B) David Gandy. Bright green = eyelid borders; cyan = eyebrows; dark green = median line and glabella; yellow = nose; white = facial contour including trichion and bilateral hairline points; dark blue = inner/outer lips. Eighty-two key landmarks were shown on each celebrity; the remaining 18 landmarks (detailed hairline, temporal fusion, etc) were not included for visibility purposes. Images reproduced with permission from Shutterstock.com.

For each subject, 100 facial landmarks (Figure 1) were detected through a custom, semi-automatic facial analysis program. The first step of this program utilizes AI facial recognition to detect basic landmarks (such as the borders of the eyebrows, nose, lips, midline of the face, and points along the peripheral facial contour). Apple, a forefront developer of AI, released a computer vision algorithm called Vision Framework that was made available to developers around the world, and we utilize this in our research to extract facial features from photographs. We combined several versions of Vision to create our desired concentration of facial landmarks in the upper, middle, and lower face. Additional custom points such as hairline, glabella, and nasal ala were added using custom-programmed MATLAB software (Mathworks, Natick, MA). Each landmark point was manually confirmed by 3 independent graders for accuracy.

**Figure 2.**
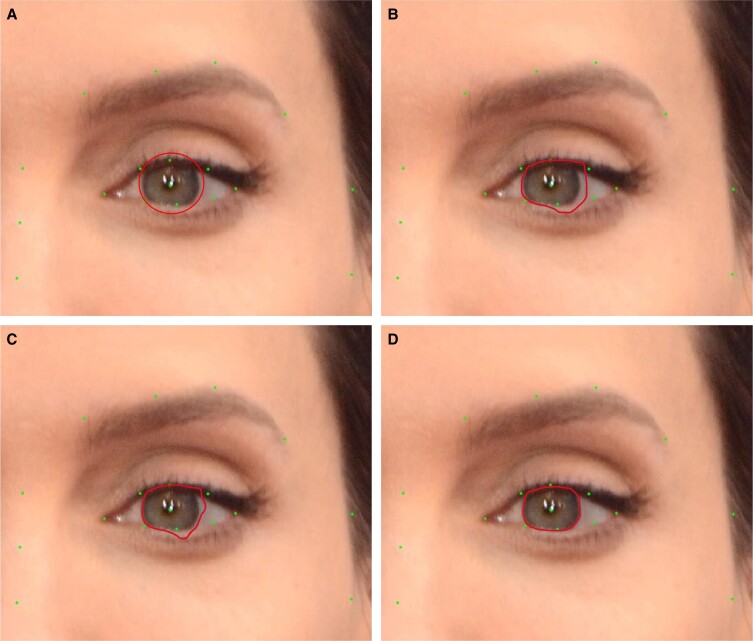
Each subject's corneal white-to-white was segmented using an automatic, active contour technique in MATLAB (Mathworks; Natick, MA). (A) Initial canthal points were utilized as pilot points to (B, C) iteratively detect white-to-white boundaries. (D) The final segmentation was then fitted to a circle to obtain final white-to-white diameter for that eye. The left and right diameters were averaged to obtain white-to-white corneal diameter for each subject. Images reproduced with permission from Shutterstock.com.

To convert pixel distances to absolute distances, each measurement in pixels was divided by the subject's white-to-white corneal diameter in pixels. Corneal white-to-white boundaries were automatically detected through an automatic, active contour technique in MATLAB (Figure 2). This technique utilized subjects’ canthus points as initial pilot points to iteratively detect our object boundaries (the pigmented iris) segment the image's foreground (a pigmented iris) from background (sclera). The final segmentation was fitted to a circle to obtain the corneal white-to-white diameter for that eye. This was also manually confirmed by 3 independent graders for accuracy, including the senior author (B.A.S.). Diameters were averaged between left and right eyes to obtain the subject's corneal white-to-white diameter in pixels. Each landmark distance in pixels was divided by the subject's white-to-white diameter in pixels. Subsequently, that ratio was multiplied by the average published white-to-white corneal diameter in millimeters, which was 11.71 ± 0.42 mm in Caucasians.^11^ This method was first validated in volunteer subjects by comparing a subset of measurements obtained using our method against manual measurements obtained from subjects’ photographs that incorporated a reference ruler for scale (*n* = 78 facial measurements across 6 white volunteer subjects). The average difference between the two measurements was 1.17 ± 1.14 mm. Paired differences between manual measurements and our method were evaluated using the Wilcoxon signed-rank test and found not to be statistically significant (*P* = .96).

To account for the rotation of the head, an overall facial rotation angle was calculated for each portrait. Facial rotation angle was calculated by finding the angle between the median face vector (line from glabella to menton) and the true vertical vector. A positive facial rotation angle indicated a clockwise rotation. To correct for any deviation from a full front-view photograph, we used the average of the corresponding right and left facial measurements for our analysis.

**Figure 3.**
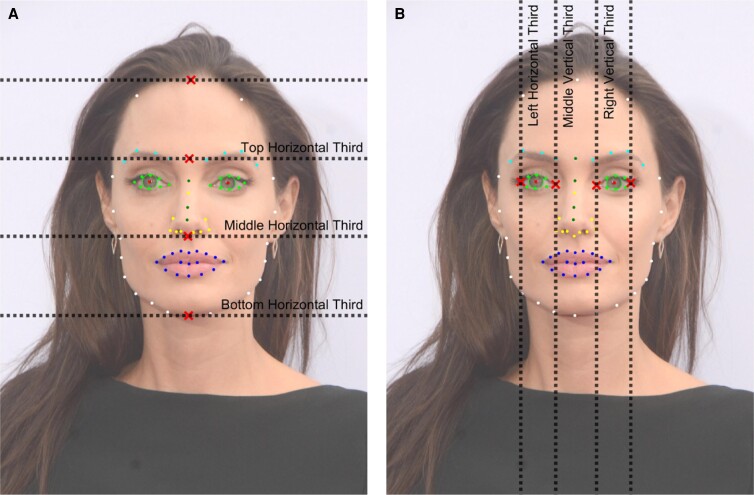
(A) Horizontal and (B) vertical facial proportions. Horizontal divisions made using the following landmarks (shown as red “x” marks on the left photograph): Trichion, Glabella, Subnasale, Menton. Vertical divisions made using the following landmarks (shown as red “x” marks on the right photograph): right exocanthion, right endocanthion, left endocanthion, left exocanthion. Images reproduced with permission from Shutterstock.com.

**Figure 4.**
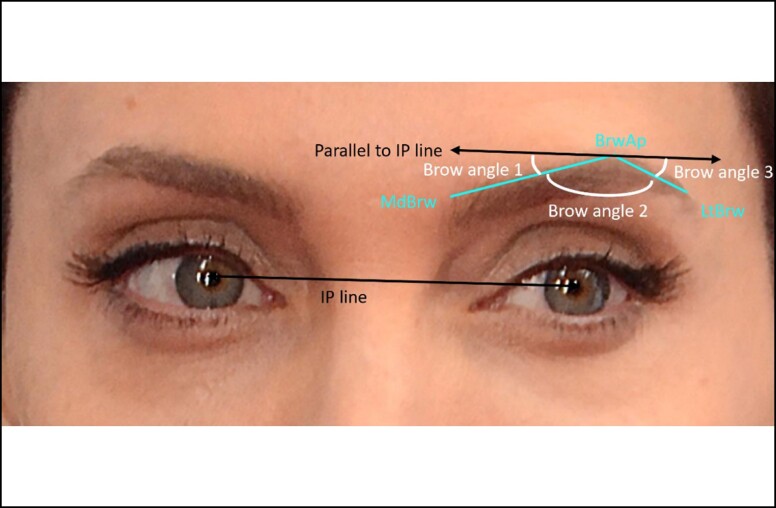
Brow measurements for Angelina Jolie. Key brow landmarks shown in cyan: MdBrw = medial brow; BrwAp = brow apex; LtBrw = lateral brow. Interpupillary line (IP line) shown in black. Line parallel to IP line also shown in black. Brow angles shown in white: Brow Angle 1 formed by MdBrw-BrwAp-Parallel to IP line; Brow Angle 2 formed by MdBrw-BrwAp-LtBrw; Brow Angle 3 formed by LtBrw-BrwAp-Parallel to IP line. Medial eyebrow length was measured as the distance between MdBrw-BrwAp. Lateral eyebrow length measured as the distance between BrwAp-LtBrw. Total eyebrow length was the sum of medial eyebrow length and lateral eyebrow length. Image reproduced with permission from Shutterstock.com.

**Figure 5.**
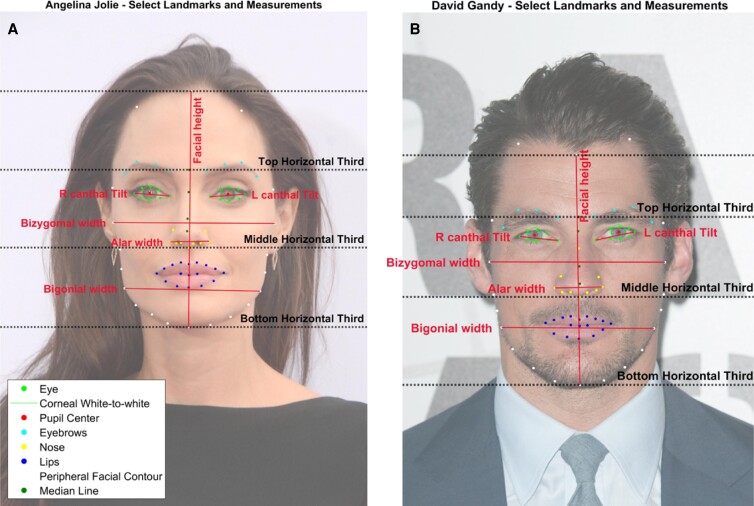
Comparison of sample facial landmarks for (A) Angelina Jolie and (B) David Gandy. Bright green = eyelid borders; cyan = eyebrows; dark green = median line and glabella; yellow = nose; white = facial contour including trichion; dark blue = inner/outer lips. Images reproduced with permission from Shutterstock.com.

From the 100 facial landmarks, the following measurements were calculated:

1. Lengths of the face
  - Total facial height (trichion to menton)
  - Face width (bizygomatic width)
2. Proportions of the face^12^
  - Horizontal thirds (Figure 3A)—Calculated both physical lengths (cm) and proportions of the overall face height (eg, a value of 0.333 for the upper face proportion would mean it was exactly 1/3 of the face height)
    - Upper face: Trichion to glabella
    - Midface: Glabella to subnasale
    - Lower Face: Subnasale to menton
  - Vertical thirds (Figure 3B)—adapted from vertical fifths.^12^ Calculated both physical lengths (cm) and proportions of the overall facial width (eg, a value of 0.333 for the middle vertical face proportion would mean it was exactly 1/3 of the distance between R exocanthion and L exocanthion)
    - R exocanthion to R endocanthion
    - R endocanthion to L endocanthion
    - L endocanthion to L exocanthion
3. Other measurements:
  - Eyebrow angles (Figure 4)
    - 3 angles from the medial eyebrow point, apex, and lateral eyebrow point were obtained.
    - Angles were measured in relation to the interpupillary line to account for the tilting of the head.
    - For each of the three angles, left and right were averaged so that each subject had three unique brow angles used for analysis.
  - Eyebrow lengths (Figure 4)
    - The brow was separated into medial and lateral portions:
      1. Medial brow (from medial brow point MdBrw to BrwAp)
      2. Lateral brow (from apex BrwAp to lateral brow point LtBrw)
      3. Total brow (MdBrw-BrwAp-LtBrw, ie, medial brow length + lateral brow length)
  - Canthal tilt
    - The angle between a line from the endocanthion to the exocanthion and a horizontal vector was calculated. For each subject, the facial rotation angle was subtracted from the canthal angles. The final canthal tilt for each subject was calculated by averaging the left and right eye canthal angles after adjusting for face rotation.
  - Bigonial width (Figure 5A, B)
  - Alar width
  - Nose height (distance from nasion to pronasale)
  - Interpupillary distance
  - Scleral show: distance from the most inferior point of iris to lower eyelid
    - If the inferior border of the iris was above the lower canthal margin, the subject was recorded to have positive scleral show.

## RESULTS

The female celebrities and models included in our study were: Angelina Jolie, Alecia Beth Moore, Ashley Graham, Bella Hadid, Catherine Zeta-Jones, Christina Applegate, Cindy Crawford, Courtney Cox, Gigi Hadid, Gisele Bundchen, Gwyneth Paltrow, Jennifer Garner, Jodie Foster, Julia Roberts, Karlie Kloss, Kate Hudson, Kendall Jenner, Michelle Pfeiffer, Nicole Kidman, Rosie Huntington, and Sandra Bullock. The male celebrities and models included in our study were: Adam Levine, Blake Shelton, Bradley Cooper, Channing Tatum, Chris Hemsworth, David Beckham, Leonardo DiCaprio, Mel Gibson, Arthur Kulkov, David Gandy, John Kortajarena, Sean O’Pry, Tobias Sorensen, Noah Mills, Ollie Edwards, Paul Rudd, Ryan Burns, Ryan Reynolds, Simon Nessman, Tom Cruise, and Tyson Ballou.

### Upper Face

#### Eyes

Females had significantly greater canthal tilt: 8.50° ± 2.10° compared to males (6.51° ± 2.67°, *P* < .010; Table 1; Figure 5A, B). Analysis of the eyes showed no significant difference in horizontal palpebral fissure length or interpupillary distance between males and females. None of the faces exhibited scleral show.

**Table 1.** Summary of Celebrity Facial Measurements

Facial measurement		Female	Male	Statistics
Facial height (cm)		17.94 ± 1.14	19.29 ± 1.24	*P* < .001***
Canthal tilt		8.50° ± 2.10°	6.51° ± 2.67°	*P* < .010***
Palpebral fissure length (cm)		2.71 ± 0.15	2.73 ± 0.21	*P* < .488
Interpupillary distance (cm)		6.17 ± 0.38	6.38 ± 0.33	*P* < .06
Eyebrow angles	1	12.43° ± 8.68°	9.90° ± 5.42°	*P* < .264
	2	51.12° ± 8.99°	48.57° ± 10.08°	*P* < .391
	3	32.45° ± 19.78°	38.67° ± 10.08°	*P* < .206
Medial eyebrow length (cm)		3.04 ± 0.27	3.39 ± 0.43	*P* < .003***
Lateral eyebrow length (cm)		1.62 ± 0.30	1.71 ± 0.29	*P* < .317
Total eyebrow length (cm)		4.66 ± 0.42	5.10 ± 0.38	*P* < .001***
Medial canthus to medial eyebrow (cm)		1.86 ± 0.33	1.73 ± 0.36	*P* < .661
Lateral canthus to lateral eyebrow (cm)		1.87 ± 0.24	1.84 ± 0.22	*P* < .204
Nose height (cm)		3.82 ± 0.34	4.17 ± 0.38	*P* < .003***
Alar width (cm)		3.21 ± 0.37	3.60 ± 0.37	*P* < .0001***
Bizygomatic width (cm)		13.05 ± 0.75	13.90 ± 0.80	*P* < .001***
Bigonial width (cm)		11.13 ± 0.70	12.34 ± 0.77	*P* < .0001***

*** Statistically significant, *P* < .05.

#### Eyebrows

Males had significantly longer medial and total brow lengths (distances AB; 3.39 ± 0.43 cm compared to females; 3.04 ± 0.27 cm, *P* < .003). In addition, males had longer eyebrows (distance AC; 5.09 ± 0.37 cm compared to females; 4.65 ± 0.42 cm). However, there were no significant differences in lateral brow lengths (distance BC), the three eyebrow angles, distances between lateral brow and lateral canthus, or medial brow to medial canthus among the genders. The proportion of the upper facial third to total facial height was significantly larger in females (*P* < .030); however, the absolute lengths of the upper facial thirds were not significantly different between sexes.

### Midface

Males had significantly longer nasal height (4.17 ± 0.38 cm) compared to females (3.82 ± 0.34 cm, *P* < .003; Table 1). Males also had greater alar widths (3.60 ± 0.37 cm) than females (3.21 ± 0.37 cm, *P* < .0001), as well as greater midfacial width (bizygomatic width; 13.90 ± 0.80 cm) than females (13.05 ± 0.75 cm, *P* < .001). The proportion of the midface length to total facial height (but not absolute midface height) was significantly greater in females (*P* < .015).

### Lower Face

Males had wider bigonial widths (12.34 ± 0.77 cm) than females (11.13 ± 0.70 cm, *P* < .0001). Both the proportion and the absolute distances of the lower facial third were significantly greater in males (*P* < .0001 and *P* < .0001, respectively).

### Facial Proportions

Males had longer faces than females (19.29 ± 1.24 vs 17.94 ± 1.14 cm, respectively). Females had a greater bizygomatic width to bigonial width ratio (*P* < .001) signifying a more tapered outer facial contour in females. Furthermore, the ratio of facial height to bigonial width was greater in females compared to males (*P* < .047, Table 2). Interestingly, the facial height to bigonial width was 1.613 in females, closely equaling the golden ratio of 1.618. That same ratio was found to be 1.566 in males, also close to the golden ratio. There was no difference in the ratio of facial height to bizygomatic width between males and females.

**Table 2.** Summary of Findings in Celebrity Facial Proportions and Ratios

Facial proportion or ratio	Female	Male	Statistics
Upper horizontal third: proportion of face height (decimal)	0.324 ± 0.023	0.308 ± 0.023	*P* < .030***
Middle horizontal third: proportion of face height (decimal)	0.344 ± 0.020	0.329 ± 0.017	*P* < .015***
Lower horizontal third: proportion of face height (decimal)	0.332 ± 0.017	0.363 ± 0.022	*P* < .0001***
Left vertical third: proportion of L to R exocanthion (decimal)	0.311 ± 0.012	0.314 ± 0.013	*P* < .559
Middle vertical third: proportion of L to R exocanthion (decimal)	0.374 ± 0.017	0.371 ± 0.020	*P* < .710
Right vertical third: proportion of L to R exocanthion (decimal)	0.315 ± 0.010	0.315 ± 0.013	*P* < .930
Ratio of facial height/bigonial width	1.613 ± 0.063	1.566 ± 0.085	*P* < .047***
Ratio of facial height/bizygomatic width	1.375 ± 0.048	1.390 ± 0.082	*P* < .488
Ratio of bizygomatic width/bigonial width	1.174 ± 0.048	1.128 ± 0.039	*P* < .001***

L, left; R, right. ***Statistically significant, *P* < .05.

## DISCUSSION

The face plays a critical role in human interactions as it is often the first anatomical part of the body through which social interactions start. There is no doubt that facial attractiveness can impact the trajectory of one's life and contribute significantly to one's self-perception,^13-16^ and one important cue for this facial attractiveness is exhibited in sexual dimorphism. A recent study by Yalcinkaya et al examined sexual dimorphism between multiracial celebrity faces and age-matched, average faces.^5^ However, a direct comparison between male and female celebrities was not made in that study, as average and celebrity female faces were grouped together and compared with the male group, which consisted of average and celebrity males. Furthermore, facial proportions and definitions of beauty are known to vary across different cultures and ethnicities.^7,17,18^

Here, we introduce a method that harnesses AI from facial recognition software to quantitatively study sexual dimorphism in Caucasian celebrity faces considered to be aesthetic. We show that this method accurately extracts and measures facial features from frontal facial photographs, and we demonstrate its utility through a focused study on Caucasian celebrities.

Our analysis showed that Caucasian male celebrities had both greater facial height and width compared to females, which supports similar findings in the average population.^4,19^ In addition, this difference in facial height between male and female celebrities derives mostly from the lower third of the face. Males exhibited a longer lower facial third, which contributed to a longer overall face height than females, despite no differences in the absolute heights of the upper and middle facial thirds. Aesthetic male faces also had greater nasal length and alar width compared to aesthetic females (*P* < .003 and *P* < .0001 respectively), which supports previous studies finding greater nostril width in average males.^4^

A previous comparison of Caucasian female celebrity faces to average female faces demonstrated that the attractive female face is smaller and is more uniformly divided into horizontal thirds and vertical fifths.^12^ Our study supported this; in aesthetic Caucasian females, facial proportions were more uniformly divided into thirds (from upper to lower; 0.324, 0.344, and 0.332, respectively), whereas in males, the ratios were less uniform (from upper to lower; 0.308, 0.329, and 0.363, respectively). Caucasian female faces had greater upper and middle third facial proportions, but males had significantly greater lower third which also corroborates previous findings.^20^ It was interesting that there was significant sexual dimorphism in facial proportions, but when looking at absolute distances in centimeters of these facial thirds, only the lower facial third was significantly greater in males (5.99 ± 0.58 cm in males vs 7.00 ± 0.58 cm in females value, *P* < .0001). This can be explained by males having a greater overall facial height than females; ie, when males’ smaller upper facial proportion is multiplied by their overall larger facial height to obtain the absolute distance, this absolute distance is similar to females’ absolute distance, which was calculated by multiplying females’ greater upper facial proportion by their smaller overall facial height. This highlights that absolute values may be less important than proportions, a consideration when planning aesthetic and FGAS.

One way to evaluate facial attractiveness is by studying facial proportions.^21-23^ A previous study comparing Caucasian female celebrities to anonymous female volunteers found that attractive female faces tend to meet criteria for ideal proportions presented in the neoclassical canons.^12^ In our study, the ratio of facial height to bigonial width was found to be 1.613 ± 0.063 (*P* < .047) in attractive females, which almost equated to the golden ratio of 1.618; traditionally used to guide conversations on aesthetic surgery and attractiveness.^24,25^ While the ratio of facial height to bigonial width was statistically less in males than in females (1.57 ± 0.09, *P* < .0470), the ratio in males, however, still approximated the golden ratio of 1.618, suggesting the importance of this ratio in contemporary facial beauty in both sexes. The decrease in this ratio in males can be explained by males having greater bigonial width relative to facial height, which supports other morphologic studies.^4,19,26^ This finding is important to note when planning surgical and nonsurgical facial rejuvenation. Widening the bigonial width with superficial musculoaponeurotic system (SMAS) manipulation, facial implants, or dermal fillers in the mandibular angle area may have a masculinizing effect on the female face. Likewise, injection of neurotoxins into the masseter muscles and bony contouring of the mandibular ramus result in a decrease in bigonial width and tapering of the lower face which feminizes the face.

There was no significant difference in the ratio of facial height to bizygomatic width between males and females. This suggests that males’ increased bigonial width may play a greater role in overall facial contour perception between sexes than their increased bizygomatic width, as supported by males having a significantly smaller ratio of bizygomatic to bigonial width (*P* < .001). As such, the outer facial contour of males tends to taper less when compared with females. This finding has important implications for aesthetic facial rejuvenation and FGAS when considering relative manipulation of the cheek and lower jaw contour and width.

Regarding eyebrow differences, males had a significantly longer medial brow portion than females (*P* < .003). Overall brow length was significantly greater in males (5.01 ± 0.38 cm) compared to females (4.66 ± 0.42 cm, *P* < .003). However, lateral brow length did not significantly differ between genders. Taken together, males have an overall greater brow length compared to females, and this greater length derives mostly from the medial portion of their brow. Previous studies have found that males’ inferior forehead region (above the eyebrow) projects more outwardly than females;^4^ our analysis was 2D in nature and thus did not look at projection. Further studies with 3-dimensional (3D) imaging systems may test the association between increased forehead projection and greater brow length. Surprisingly, there were no significant differences in eyebrow angles 1, 2, and 3 between males and females. Furthermore, there was no difference among genders in medial brow to medial canthus distance or in lateral brow to lateral canthus distance. However, females had 2° more canthal tilt compared to males (*P* < .010), indicating that females have a more angular canthal shape independent of their brow angles. Both genders had greater canthal tilt (8.50° ± 2.10° in females, 6.51° ± 2.67° in males, *P* < .010) than that found in general populations (4.12° in the average Caucasian face),^27^ suggesting that greater canthal tilt for males and females is considered to be more attractive. Palpebral fissure length and interpupillary distance were not significantly different in the attractive male and female faces analyzed, similar to the lack of sexual dimorphism seen in the general Caucasian population.^19^

### Limitations

Our study was limited by the use of 2D photographs that do not provide as much information as 3D face scanners. Having 3D data on celebrities would be helpful in identifying topographic differences between males and females, but such scans would be difficult to acquire. Although we were careful in curating frontal, nonanimated photographs of all subjects and corrected for rotation of the head in the XY plane, there may have been subtle changes in rotation of the head along other axes. Despite these limitations, we believe that our study provides novel methods and relevant insights into aesthetic and gender affirming procedures.

## CONCLUSIONS

This is the first study using artificial intelligence software to accurately quantify sexual dimorphisms between male and female Caucasian faces considered attractive. Aesthetic female faces had greater upper and middle proportions of the face, steeper canthal tilt, more uniform facial thirds, more tapered facial contour from malar to gonial region, slimmer alar width, and ratio of facial height to bigonial width equating to the golden ratio of 1.618. Aesthetic male faces had greater lower facial third proportion, greater facial height, and width, increased alar width, increased nasal length, greater medial and overall eyebrow length, and less angular canthal tilt. Palpebral fissure, interpupillary distance, eyebrow angles, and ratio of facial height to bizygomatic width were similar between genders. This proof-of-concept method may be expanded to analyzing facial sexual dimorphism in other ethnicities and the general population which may help guide clinicians in providing inclusive and culturally sensitive, evidence-based aesthetic assessment and parameters when undergoing facial rejuvenation and gender-affirming surgery.
