# Correction to: Using Artificial Intelligence to Quantify Sexual Dimorphism in Aesthetic Faces: Analysis of 100 Facial Points in 42 Caucasian Celebrities

**DOI:** 10.1093/asjof/ojad097

**Published:** 2023-11-06

This is a correction to: Alice S Liu, BS, Cristina A Salinas, BS, Basel A Sharaf, MD, “Using Artificial Intelligence to Quantify Sexual Dimorphism in Aesthetic Faces: Analysis of 100 Facial Points in 42 Caucasian Celebrities,” *Aesthetic Surgery Journal Open Forum*, Volume 5, 2023, ojad046, https://doi.org/10.1093/asjof/ojad046.

In the originally published version of this manuscript, the author regrets that there was an error in the original version of Table 1 regarding the average interpupillary distance in males. The corrected version of the table is included here and has been corrected online in the version of record.

